# Interaction of ^3^H^+^ (as HTO) and ^36^Cl^−^ (as Na^36^Cl) with crushed granite and corresponding fracture infill material investigated in column experiments

**DOI:** 10.1007/s10967-013-2870-7

**Published:** 2013-12-13

**Authors:** K. Štamberg, Š. Palágyi, K. Videnská, V. Havlová

**Affiliations:** 1 Department of Nuclear Chemistry, Faculty of Nuclear Sciences and Physical Engineering, Czech Technical University, 115 19 Prague, Czech Republic; 2 ÚJV Řež, a.s., Husinec-Řež, Řež, Czech Republic; 3 Department of Analytical Chemistry, Institute of Chemical Technology, 166 28 Prague 6, Czech Republic

**Keywords:** Crushed granite, Groundwater, HTO, Na^36^Cl, Dynamic conditions, Modeling

## Abstract

The transport of ^3^H^+^ (as HTO) and ^36^Cl^−^ (as Na^36^Cl) was investigated in the dynamic system, i.e., in the columns filled with crushed pure granite and fracture infill of various grain sizes. The aim of column experiments was to determine important transport parameter, such as the retardation, respectively distribution coefficients, Peclet numbers and hydrodynamic dispersion coefficients. Furthermore, the research was focused to quantification of the effect of grain size on migration of studied radionuclides. The experimental breakthrough curves were fitted by a model based on the erfc-function, assuming a linear reversible equilibrium sorption/desorption isotherm, and the above mentioned transport parameters were determined. The results showed that influence of grain size on sorption of ^3^H^+^ and ^36^Cl^−^ was negligible. Retardation and distribution coefficients of both tracers converged to one and zero, respectively, in case of all fractions of crushed granite and infill material. Generally, the presumed ion exclusion of ^36^Cl in anionic form was proved under given conditions, only very weak one seems to exist in a case of infill material. In principal, both radionuclides behaved as non-sorbing, conservative tracers. On the other hand, the influence of grain size on Peclet numbers value and on dispersion coefficient was observed for both crystalline materials, namely in agreement with theoretical suppositions that the values of Peclet numbers decrease with increasing grain size and values of dispersion coefficient increase.

## Introduction

The granitic rock is investigated as a potential host rock, being considered as one of the safety barriers for high-level radioactive waste disposal in number of countries, including Czech Republic. Therefore, the ability of host rock to retard radionuclide migration is an important property. Migration of radionuclides describes the transport parameters such as the retardation and distribution coefficients (*R* and *K*_d_), Peclet number (*Pe*) or hydrodynamic dispersion coefficient (*D*) (in a case of 2D- or 3D-model it deals with longitudinal hydrodamic dispersion coefficient). These parameters can be determined using dynamic column technique, which has many advantages in comparison with static batch experiments. Namely, it enables: (i) the direct determination of contaminant retardation coefficients dissolved in aqueous phase, (ii) the closer approximation of the real conditions in the natural system, (iii) the study of less disturbed samples, and, (iv) the study of desorption process following immediately after sorption, and so on. This all can clarify the behavior of contaminants in given natural system [1, 2].

Results of column experiments with such type of radionuclides can provide information about the properties of the solid phase in the column. Namely, the values of Peclet numbers, hydrodynamic dispersion coefficient and accessible porosity can be obtained from the peak or the whole breakthrough curve. Radionuclides ^3^H^+^ (in the form of tritiated water, HTO) and ^36^Cl^−^ (in the form of Na^36^Cl) are regarded as non-interacting (*K*_d_ → 0, *R* → 1) conservative tracers. Moreover, anionic exclusion is usually under consideration for ^36^Cl^−^. On the other hand, dynamic experiments with these radionuclides can be therefore used to describe hydrodynamic characterization of the given column [3, 4]. For example, HTO as conservative tracer was used within a series of column experiments with colloids for comparison retardation time of colloids and studying radionuclides (e.g., ^244^Pu, ^241^Am, ^152^Eu). The experiments with HTO were conducted at different flow rates [4, 5]. Voudrias et al. studied transport of tritium in mudstone, halite and carbonate rocks by column sorption/desorption experiments. In this case, the experimental tritium breakthrough curves were fitted using the analytical solution of 1-D ADE (advection–dispersion equation) and the best-fit Peclet numbers were used to calculate the column dispersivity. The results showed that dispersion was very low [6]. Tritiated water was also used for the study of nanoparticles transport through fractured crystalline rock. The shape of HTO and nanoparticles breakthrough proved the influence of fracture heterogeneity on flow velocity distribution and on mass transport [7]. Moreover, tritiated water was used in laboratory column experiment in order to illustrate the anionic exclusion effect of anionic tracers (SO_4_^2−^, I^−^) in diffusion tests, and to estimate the iodine sorption on argillites and limestone [8]. Then, HTO was used to investigate the influence of column lengths and flow rates on transport of radionuclides in crushed granite. Results of experimental and numerical investigations showed that in a case of short column the dispersivity was overestimated and so the retardation factor was underestimated. Experiments with HTO therefore pointed out the necessity of using long column and comparing experimental HTO breakthrough curves with numerical simulations before start of the experiments [9].

Transport and retardation of HTO and ^36^Cl^−^ in crystalline rock were widely studied by Hölttä et al. They focused on transport through sawed fracture column and natural fracture column. The results showed that hydrodynamic dispersion is process dominating in transport of conservative radionuclides in sawed fracture column. The differences between elution time of tritium and chlorine were not observed. Experiments with natural fracture column showed higher retardation and dispersion of tritiated water in comparison with chlorine, probably due to ion-exclusion of the anions in fracture infill [10, 11]. Effect of matrix on transport of chlorine and tritium was observed in case of lower flow rates. On the other hand, advection was dominant process at the fastest flow rates of liquid phase [12].

Mathematical models used to the modeling of radionuclide migration generally are based on the analytical solution of 1-D advection–dispersion equation. Such models include all partial processes which can participate in transport process studied, such as dispersion, convection, interaction characterized with sorption/desorption linear isotherm equation, radioactive decay and so on, see e.g. [13]. There are also such models at hand [14–17] making possible to describe the interaction characterized not only with linear, but also with non-linear isotherm equation, i.e., if the retardation and distribution coefficients are function of investigated component concentration—of course, it is rarely a case of the non-interacting tracers type of HTO and ^36^Cl^−^.

The present work investigated migration of tracers HTO and ^36^Cl^−^ (as Na^36^Cl) in columns filled with crushed pure granite and corresponding infill materials. Four fractions (from each type of material), having different grain size, were used as a solid phase and synthetic granitic groundwater as a liquid phase. Experiments were focused on the study of the effect of grain size, firstly on the values of Peclet numbers, hydrodynamic dispersion coefficient and bed porosity. Secondly, the influence of grain size on the values of retardation and distribution coefficients was followed. The transport model with incorporated linear isotherm equation was used to the description and modeling of experimental data.

## Experimental

Analogous to the previous studies [16–18], two following types of crystalline rocks were used: pure granite, coded as PDM1-1, and fracture infill material, coded as PDM1-2. Rocks were sampled from PDM1 borehole; the samples PDM1-1 from 97.5 to 98.7 m depth and PDM1-2 from 89.7 to 90.0 m depth. The X-ray phase analysis of granitic materials is presented in Table 1 [15]. Each rock sample was crushed and sieved to 0.063–0.125, 0.125–0.630, 0.63–0.80 and 0.80–1.25 mm fractions. The fractions were placed into 5 cm^3^ adapted plastic columns of 1.3 cm inner diameter and 5.4 cm height. The basic hydraulic parameters of column filled with crushed rocks (bulk density, porosity and pore volume) were calculated using experimentally measured values of mass and total volume of the column bed and on the assumption that specific weight of both borehole samples equals 2.67 g/cm^3^ (see, e.g., Table 2).

**Table 1:** X-ray analysis mineral phases for crushed pure granite (PDM1-1) and fracture infill material (PDM1-2) (average values of mineral content and corresponding standard deviations, SD, given in %) [15]

Sample	Majority phase			Minority phase	
	Quartz	Orthoclase	Plagioclase	Mica	Chlorite
PDM1-1^a^	31 ± 1	33 ± 3	21 ± 3	11 ± 1	5 ± 1
PDM1-2^b^	35 ± 2	25 ± 2	26 ± 1	5 ± 1	10 ± 1

^a^Traces of augite, hematite and calcite

^b^Traces of kaolinite

**Table 2:** Column parameters for ^3^H^+^ (as HTO) filled with crushed pure granite (PDM1-1a,b,c,d) and fracture infill material (PDM1-2a,b,c,d). Bed volume (V): 6.4 cm^3^ and bed height (L): 5.4 cm (2.5–3 pore volumes of liquid phase, *n*_PV_, were applied in the course of each experiment under the given seepage velocity)

Column no.	Grain size (mm)	Weight (g)	Bulk density (*ρ*) (g/cm^3^)	Porosity (*θ*) (cm^3^/cm^3^)	Pore volume (PV) (cm^3^)	SGW seepage velocity (*u*) cm/min
PDM1-1a	0.063–0.125	8.525	1.332	0.501	3.51	0.072
PDM1-2a	0.063–0.125	8.531	1.333	0.501	3.51	0.070
PDM1-1b	0.125–0.63	9.640	1.506	0.436	3.09	0.080
PDM1-2b	0.125–0.63	9.585	1.498	0.439	3.11	0.076
PDM1-1c	0.63–0.80	9.544	1.491	0.441	3.13	0.081
PDM1-2c	0.63–0.80	9.490	1.483	0.448	3.15	0.082
PDM1-1d	0.80–1.25	9.540	1.491	0.444	3.13	0.085
PDM1-2d	0.80–1.25	9.560	1.494	0.448	3.12	0.087

Tracer solution was obtained adding appropriate aliquot of HTO or Na^36^Cl (with high radiochemical purity, min. 99 %) into a defined volume of the synthetic groundwater (SGW). Its composition is in Table 3 [14]. The experimental setup and procedure of the experiments can be found in [16, 17]. The initial activity and flow-rate about 0.05 cm^3^/min of SGW through the columns were constant during the sorption process. Three-cm^3^ samples of liquid phase were taken in selected time intervals from a separated volume at the column outlet for beta activity measurement. The separated volume and the overall flowed out volume of liquid phase were recorded. After reaching the steady state, SGW without HTO or Na^36^Cl was used for radionuclide desorption from the column at the same flow rate until new steady state had been reached. The sampling of SGW and the activity measuring were made in the same way as before.

**Table 3:** Composition of synthetic granitic groundwater (SGW) [14]

Component	mg/dm^3^
Na^+^	10.6
K^+^	1.8
Ca^2+^	27.0
Mg^2+^	6.4
Cl^−^	42.4
SO_4_^2+^	27.7
NO_3_^−^	6.3
HCO_3_^−^	30.4
F^−^	0.2
pH	8.3

## Transport model

The transport model is based on erfc-function obtained as a result of analytical solution of a 1-D advection–dispersion equation (ADE) under sorption/desorption boundary conditions [15–18]. It can be used for fitting the experimental dynamic data and for calculation (description) of a breakthrough curve (BTC). The BTC is a dependence of the output relative activity (*A*_rel_) or concentration (*C*_rel_) of the dissolved component on the number of bed pore volumes (*n*_PV_). In principle, the transport model itself can be modified by incorporation of linear equilibrium isotherm (linear isotherm approach), or non-linear equilibrium isotherm (non-linear isotherm approach), which can be found in our previous publications [14–18]. Therefore, only the basic principles of linear isotherm approach, characterizing the interaction of components type of ^3^H^+^ and ^36^Cl^−^, are given below

Theoretical sorption breakthrough curve (BTC_s_) was calculated by Eqs. (1a) and (1b). These equations were derived with assumption of reversible linear sorption isotherm [see Eq. (3)]. It holds for the theoretical relative output activity of the liquid phase from the column:

1a $$ A_{\text{relStheor}} = \frac{{A_{\text{tStheor}} }}{{A_{0} }} = 0.5 \cdot {\text{erfc}}\left[ {\frac{{R_{\text{Stheor}} - n_{\text{PVS}} }}{{2 \cdot \left( {R_{\text{Stheor}} \cdot n_{\text{PVS}} /Pe} \right)^{0.5} }}} \right] $$

1b $$ A_{\text{relStheor}} = \frac{{A_{\text{tStheor}} }}{{A_{0} }} = 1 - \left\{ {0.5 \cdot {\text{erfc}}\left[ {\frac{{ - \left( {R_{\text{Stheor}} - n_{\text{PVS}} } \right)}}{{2 \cdot \left( {R_{\text{Stheor}} \cdot n_{\text{PVS}} /Pe} \right)^{0.5} }}} \right]} \right\} $$

where:

2 $$ R_{{S{\text{theor}}}} = 1 + \frac{{\rho + K_{\text{dStheor}} }}{\theta } $$

3 $$ q = K_{\text{dStheor}} \cdot C $$

Here denotes: *A*_0_—input activity of liquid phase on the top of the column (cpm), *A*_tStheor_—theoretical value of output activity of liquid phase leaving the column in the course of sorption at time *t*_S_ (cpm), *n*_PVS_ (= *u·t*_*S*_/*L*)—experimental value of number of bed pore volumes in the case of sorption at time *t*_S_, *t*_S_—time of sorption experiment (e.g., h), *erfc—*complementary error function, *R*_Stheor_—theoretical sorption retardation coefficient, *Pe*—*Pe* (= *u*·*L*/*D*_*d*_) of the column, *u*—water seepage velocity (cm/min), *L*—length of the bed in the column (cm), *D*_d_—hydrodynamic dispersion coefficient (cm^2^/min), *K*_dStheor_—theoretical sorption distribution coefficient (cm^3^/g), *q* and *C*—total concentration of given component (e.g., NaCl) in solid (mmol/g), and liquid (mmol/cm) phase, respectively, *ρ*—bulk density (g/cm), *θ*—porosity (cm^3^/cm). It should be noted that for the calculation of *A*_relStheor_, the Eq. (1a) can be used directly until *A*_relStheor_ ≤ 0.5, i.e., until (*R*_Stheor_ − *n*_PVS_) ≥ 0, but for the calculation of *A*_relStheor_ > 0.5, i.e. if (*R*_Stheor_ − *n*_PVS_) < 0, the Eq. (1b) should be used, which was derived from Eq. (1a) (because it holds: *erfc (*−*x)* = 2 − *erfc(x)*). For the calculation of the theoretical desorption breakthrough curve (BTC_*D*_ *)*, which is centrally symmetric to the sorption BTC_*S*_, the following Eqs. (4a) (if *A*_relDtheor_ ≥ 0.5) and (4b) (if *A*_relDtheor_ < 0.5) were used, respectively:

4a $$ A_{\text{relD,theor}} = \frac{{A_{{tD , {\text{theor}}}} }}{{A_{0} }} = 1 - \left\{ {0.5 \cdot {\text{erfc}}\left[ {\frac{{R_{{D , {\text{theor}}}} - n_{\text{PVD}} }}{{2 \cdot (R_{{D , {\text{theor}}}} \cdot n_{\text{PVD}} /Pe )^{0.5} }}} \right]} \right\} $$

4b $$ C_{\text{relD,theor}} = \frac{{C_{{tD , {\text{theor}}}} }}{{C_{0} }} = 1 - \left( {1 - \left\{ {0.5 \cdot {\text{erfc}}\left[ {\frac{{ - \left( {R_{{D , {\text{theor}}}} - n_{\text{PVD}} } \right)}}{{2 \cdot \left( {R_{{D , {\text{theor}}}} \cdot n_{\text{PVD}} /Pe} \right)^{0.5} }}} \right]} \right\}} \right) $$

where:

5 $$ R_{\text{Dtheor}} = 1 + \frac{{\rho + K_{\text{dDtheor}} }}{\theta } $$

6 $$ q = K_{\text{dDtheor}} \cdot C $$

The meaning of the symbols by analogy, i.e., if index *S* (denotes sorption) is substituted by index *D* (denotes desorption), is the same as in previous equations.

The obtained sorption BTC_*S*_ has a well-known S-shape, the desorption BTC_*D*_ has a reverse shape and is mirror-symmetric to the former, both of them with the inflexion point (see index *i*) at the position where for experimental values of retardation coefficients, it holds: *R*_Sexp_ = *n*_PVSi_ or *R*_Dexp_ = *n*_PVDi_. This generally occurs at *A*_rel_ = 0.5·*A*_0_. It is evident that to the values of *R*_Sexp_ or *R*_Dexp_ the values of *K*_dSexp_ or *K*_dDexp_ correspond, respectively, which can be calculated by means of modified Eqs. (2) or (5), generally by relation *K*_dexp_ = (*R*_exp_ − 1)·*θ*))/*ρ*—of course, if *R*_exp_ ≥ 1.

The evaluation of experimental data, i.e., *A*_relSexp_ = *A*_tSexp_/*A*_0_ = f(*n*_PVS_) and *A*_relDexp_ = *A*_tDexp_/*A*_0_ = f(*n*_PVD_), by means of Eqs. (1a), (1b), (2) and (3), and Eqs. (4a), (4b), (5) and (6), respectively, consisted in their simultaneous fitting in iteration cycle by the Newton–Raphson multidimensional method of non-linear regression, in the course of which the values of three parameters, namely, *K*_dS_, *K*_dD_ and *Pe* were sought [19]. (These are the basic properties of breakthrough curves based on the error and/or complementary error functions.)

As fitting criterion, the quantity of weighted sum of squares divided by the degrees of freedom (WSOS/DF) [20] was calculated; the agreement (the goodness-of-fit) can be regarded as acceptable if 0.1 ≤ WSOS/DF ≤ 20. The respective computational code PNLRPa11.fm (Code Package Stamb-2010) was constructed for FAMULUS software product [21], which was used for calculation.

## Results and discussion

**Fig. 1:**
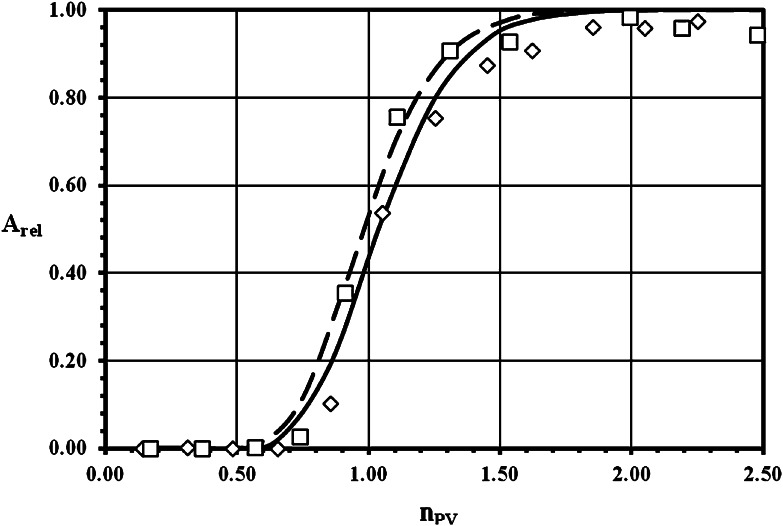
Sorption breakthrough curves for HTO in crushed pure granite (*open diamond*) and in infill material (*open square*) of grain size 0.063–0.125 mm. Symbols: experimental data, lines: calculated values (*solid line*—to (*open diamond*), *dash line*—to (*open square*)) (*A*_rel_ is the relative activity of ^3^H^+^ (as HTO) outgoing from the column and *n*_PV_ is the number of pore volumes.)

**Fig. 2:**
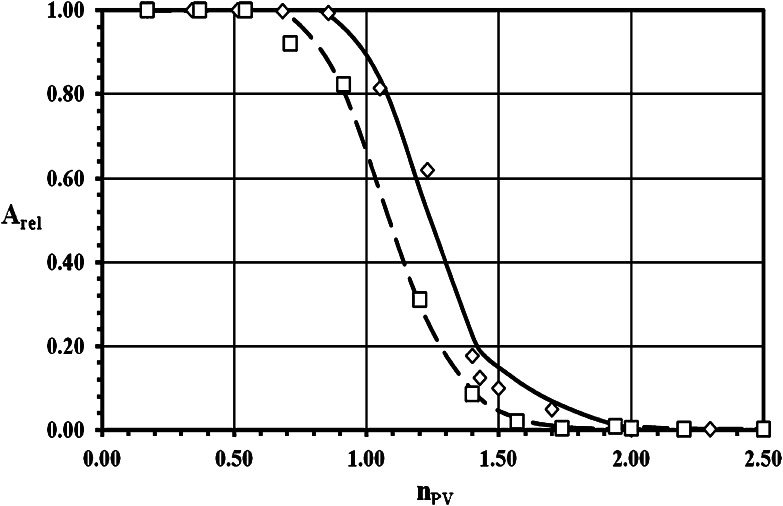
Desorption breakthrough curves for HTO in crushed pure granite (*open diamond*) and in infill material (*open square*) of grain size 0.063–0.125 mm. Symbols: experimental data, lines: calculated values (*solid line*—to (*open diamond*), *dash line*—to (*open square*)) (*A*_rel_ is the relative activity of ^3^H^+^ (as HTO) outgoing from the column and *n*_PV_ is the number of pore volumes.)

**Fig. 3:**
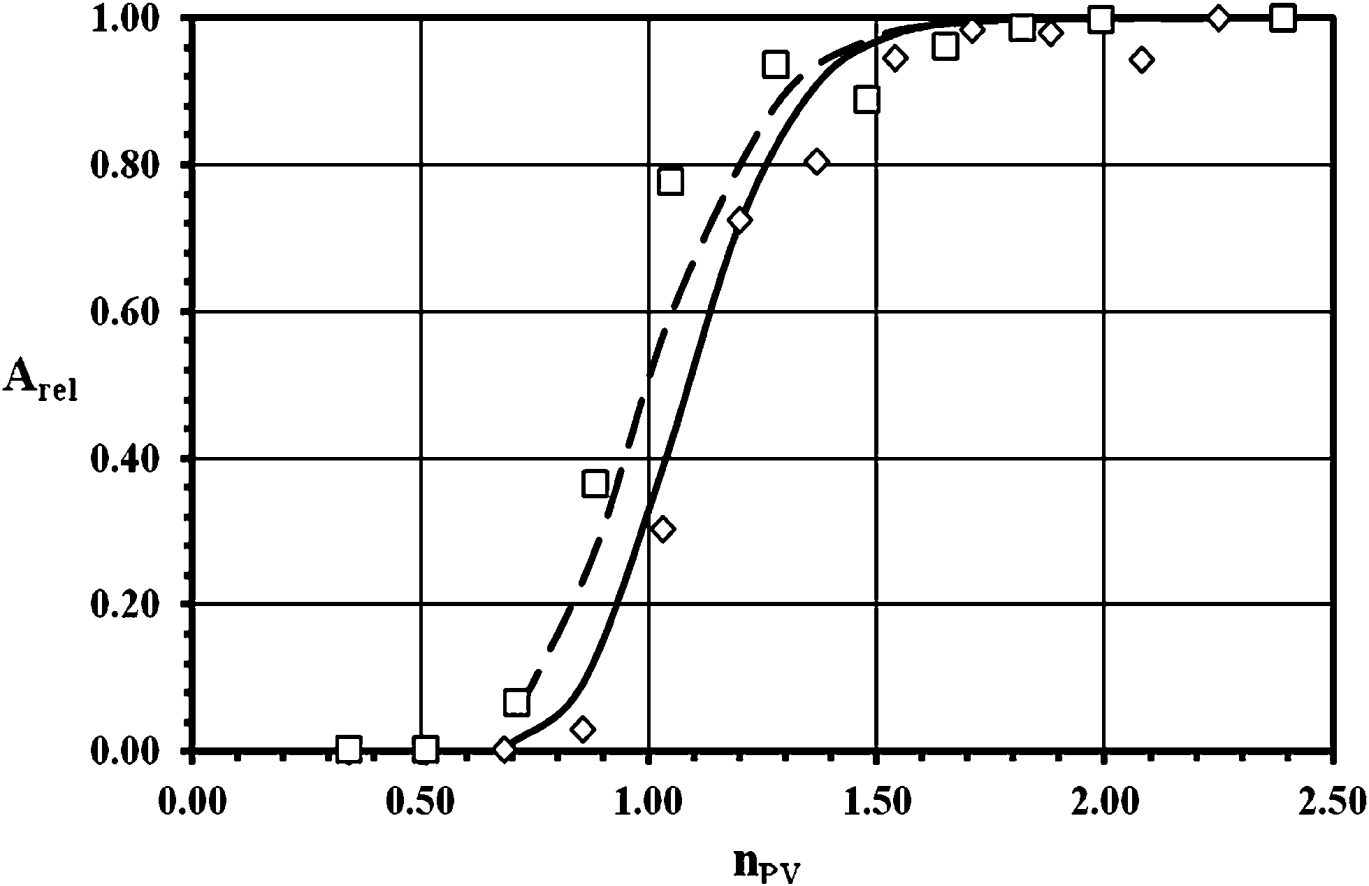
Sorption breakthrough curves for ^36^Cl^−^ (as Na^36^Cl) in crushed pure granite (*open diamond*) and in infill material (*open square*) of grain size 0.063–0.125 mm. Symbols: experimental data, lines: calculated values (*solid line*—to (*open diamond*), *dash line*—to (*open square*)) (*A*_rel_ is the relative activity of ^36^Cl^−^ (as Na^36^Cl) outgoing from the column and *n*_PV_ is the number of pore volumes.)

**Fig. 4:**
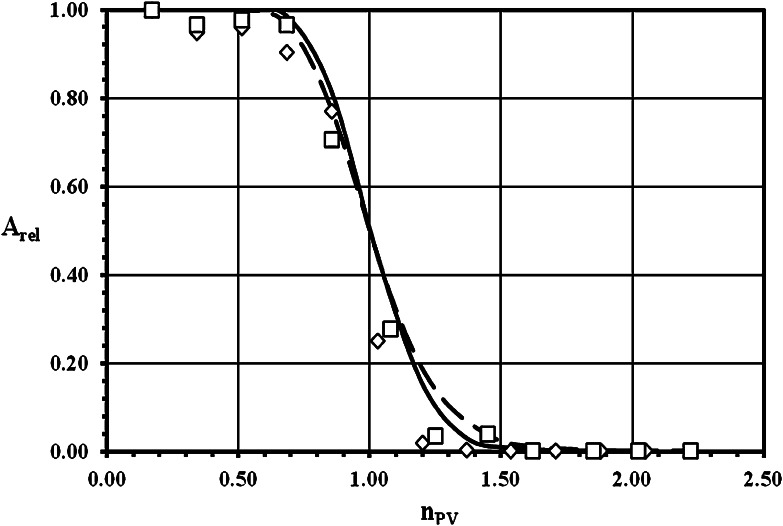
Desorption breakthrough curves for ^36^Cl^−^ (as Na^36^Cl) in crushed pure granite (*open diamond*) and in infill material (*open square*) of grain size 0.063–0.125 mm. Symbols: experimental data, lines: calculated values (*solid line*—to (*open diamond*), *dash line*—to (*open square*)) (*A*_rel_ is the relative activity of ^36^Cl^−^ (as Na^36^Cl) outgoing from the column and *n*_PV_ is the number of pore volumes.)

The experimental breakthrough curves (BTC) of ^3^H^+^ (as HTO) and ^36^Cl^−^ (as Na^36^Cl) obtained both with crushed granite and their infill materials, as well as the results of the fitting of these data with theoretical BTC calculated by linear isotherm approach model, are demonstrated for grain size 0.063–0.125 mm in Figs. 1, 2 for sorption and desorption of ^3^H^+^, respectively, and in Figs. 3, 4 for sorption and desorption of ^36^Cl^−^, respectively. The breakthrough curves for other grain sizes are similar or practically the same. This similarity is evident from (Tables 2, 4, 5 and 6), where the important transport parameters are summarized, including the values of goodness-of-fit criterion declaring the very good agreement between experimental and calculated data. Unfortunately, a few of column experiments with HTO, see PDM1-1a (Fig. 2) and PDM1-2c sorption, and with ^36^Cl^−^, see PDM1-1d and PDM1-2d, evidently are loaded with experimental error probably as a result of short columns and of the problems connected with the starting point (*n*_PV_ = 0) determination.

**Table 4:** Transport and sorption/desorption parameters of HTO in columns filled with crushed pure granite (PDM1-1) and fracture infill material (PDM1-2)—calculated using linear isotherm approach model (SD—absolute standard deviation)

Column no.	R_Sexp_	R_Dexp_	*R*_Steor_	*R*_Dteor_	*K*_dStheor_ ± SD (cm^3^/g)	*K*_dDtheor_ ± SD (cm^3^/g)	Pe_S_ ± SD	*D*_dS_ (cm^2^/min)	WSOS/DF
PDM1-1a	1.04	1.20	1.07	1.23	20E−3 ± 6E−3	89E−3 ± 5E−3	125.0 ± 12.8	3.11E−3	0.40
PDM1-2a	0.98	1.09	1.00	1.09	1E−5 ± 3E−3	31E−3 ± 3E−3	41.0 ± 4.2	9.22E−3	0.10
PDM1-1b	1.13	0.89	1.05	1.00	14E−3 ± 7E−3	4E−5 ± 8E−3	74.3 ± 11.8	5.81E−3	0.86
PDM1-2b	0.91	0.79	1.02	1.00	5E−3 ± 2E−2	5E−5 ± 4E−2	46.5 ± 14	8.82E−3	2.08
PDM1-1c	1.10	0.97	1.07	1.00	20E−3 ± 5E−3	3E−6 ± 5E−3	47.6 ± 10.7	9.19E−3	0.50
PDM1-2c	1.10	0.90	1.27	1.00	81E−3 ± 6E−3	4E−6 ± 4E−3	25.0 ± 4.2	1.77E−2	0.36
PDM1-1d	0.98	0.95	1.00	1.00	1E−5 ± 5E−3	3E−5 ± 5E−3	16.5 ± 4.3	2.78E−2	0.34
PDM1-2d	0.99	1.04	1.00	1.04	5E−6 ± 3E−3	8E−3 ± 3E−3	27.9 ± 3.3	1.68E−2	0.11
Mean values ± SD									
PDM1-1	1.06 ± 0.06	1.00 ± 0.13	1.05 ± 0.03	1.06 ± 0.12	1.3E−2 ± 9E−3	2.2E−2 ± 4E−2	–	–	–
PDM1-2	0.99 ± 0.08	0.96 ± 0.14	1.07 ± 0.13	1.03 ± 0.04	2.1E−2 ± 4E−2	1.0E−2 ± 2E−2	–	–	–

**Table 5:** Column parameters filled with crushed pure granite (PDM1-1a,b,c,d) and fracture infill material (PDM1-2a,b,c,d) for ^36^Cl^−^ (as Na^36^Cl). Bed volume (V): 6.4 cm^3^ and bed height (L): 5.4 cm (2.5–3 pore volumes of liquid phase, *n*_PV_, were applied in the course of each experiment under the given seepage velocity)

Column no.	Grain size (mm)	Weight (g)	Bulk density (*ρ*) (g/cm^3^)	Porosity (*θ*) (cm^3^/cm^3^)	Pore volume (PV) (cm^3^)	SGW seepage velocity (*u*) (cm/min)
PDM1-1a	0.063–0.125	8.5249	1.332	0.501	3.51	0.079
PDM1-2a	0.063–0.125	8.5305	1.333	0.501	3.51	0.070
PDM1-1b	0.125–0.63	9.6400	1.506	0.436	3.09	0.080
PDM1-2b	0.125–0.63	9.5851	1.498	0.439	3.11	0.076
PDM1-1c	0.63–0.80	9.5437	1.491	0.441	3.13	0.081
PDM1-2c	0.63–0.80	9.4902	1.483	0.445	3.15	0.082
PDM1-1d	0.80–1.25	9.5400	1.491	0.442	3.13	0.085
PDM1-2d	0.80–1.25	8.5249	1.332	0.501	3.51	0.087

**Table 6:** Transport and sorption/desorption parameters of ^36^Cl^−^ (as Na^36^Cl) in columns filled with crushed pure granite (PDM1-1) and fracture infill material (PDM1-2)—calculated using linear isotherm approach model (SD—absolute standard deviation)

Column no.	R_*S*exp_	R_Dexp_	*R*_Steor_	*R*_Dteor_	*K*_dStheor_ ± SD (cm^3^/g)	*K*_dDtheor_ ± SD (cm^3^/g)	Pe_S_ ± SD	*D*_dS_ (cm^2^/min)	WSOS/DF
PDM1-1a	1.11	0.94	1.08	1.00	30E−3 ± 6E−3	4E−5 ± 6E−3	86.5 ± 6.2	5.24E−3	0.69
PDM1-2a	0.94	0.96	1.00	1.00	7E−5 ± 6E−3	3E−6 ± 6E−3	69.6 ± 10.5	6.9E−3	0.14
PDM1-1b	1.08	0.92	1.03	1.00	10E−2 ± 11E−2	4E−5 ± 7E−3	27.4 ± 2.4	1.81E−2	1.25
PDM1-2b	0.86	0.91	1.00	1.05	9E−5 ± 9E−3	13E−3 ± 9E−3	31.4 ± 1.8	1.62E−2	0.10
PDM1-1c	1.08	1.06	1.13	1.06	40E−3 ± 3E−3	18E−3 ± 2E−3	27.4 ± 2.08	1.77E−2	0.15
PDM1-2c	0.84	0.92	1.00	1.01	3E−5 ± 2E−2	3E−3 ± 1E−2	25.3 ± 2.7	2.09E−2	0.24
PDM1-1d	1.09	0.92	1.30	1.11	89E−3 ± 7E−3	33E−3 ± 9E−3	41.4 ± 5.0	1.13E−2	1.20
PDM1-2d	0.98	0.93	1.23	1.06	67E−3 ± 5E−3	17E−3 ± 6E−3	30.7 ± 2.6	1.86E−2	0.46
Mean values ± SD									
PDM1-1	1.09 ± 0.01	0.96 ± 0.07	1.13 ± 0.12	1.06 ± 0.12	6.5E−2 ± 3.5E−2	1.3E−2 ± 1.6E−2	–	–	–
PDM1-2	0.90 ± 0.07	0.93 ± 0.02	1.06 ± 0.12	1.03 ± 0.03	7.7E−2 ± 3.3E−2	8.2E−3 ± 8E−3	–	–	–

The values of retardation and distribution coefficients reflect the interaction of studied tracers with the surface of given materials. As for their theoretical quantities calculated using model Eqs. (1a)–(6), it has to be taken into account that in a case of weak- or non-interacting tracers the values of retardation coefficients converge to 1 from the right (i.e., are equal or greater than 1) and distribution coefficients converge to zero from the right, too. According to the values of *R*_Stheor_ and *R*_Dtheor_, and *K*_dStheor_ and *K*_dDtheor_, especially to their mean values in Table 4 and Table 6, these conditions are better fulfilled by HTO than by ^36^Cl^−^; it is true especially, if the maximal values (probably in consequence of experimental error mentioned above) are omitted.

As for the possible ion exclusion of chloride, it exists if the value of a given retardation coefficient is smaller than 1. Evidently, such coefficient cannot be found by means of model used, and therefore, the experimental value, *R*_*Sexp*_ or *R*_*Dexp*_, has to be taken into consideration. (It deals with quantities, the meaning of which is defined above, namely, *R*_Sexp_ **=** *n*_PVSi_ or *R*_Dexp_ **=** *n*_PVDi_). These values can be found in Tables 4 and 6, and we see that especially the mean values (±SD) demonstrate, if any, only a weak ion exclusion of ^36^Cl^−^ in the case of infill material. To give an account of this phenomenon, the negative surface charge of given solid phase particles has to be supposed under experimental conditions, i.e., especially for SGW having pH 8–8.5 (see Table 3) when, more or less, the deprotonation of surface edge sites (and the creation of negative charge) can proceed. Unfortunately, on the basis of the X-ray analysis of mineral phases (see Table 1) we can only speculate about the possible contribution of, e.g., chlorite and kaolinite, to the formation of the negative surface charge of individual infill material particles. But, on the other hand, this assumption seems to be real because there are similar results in the matter of the possible ion exclusion of the anions in fracture infill material column, which can be found in papers by Hölttä et al. [10, 11] discussed in “Introduction”.

The values of Peclet numbers, summarized in Tables 4 and 6, characterizing the flow pattern (flow character in the bed), are evidently a function of the grain size. It corresponds well with the theory [22, 23], according to which such phenomenon generally depends on: (i) bed height, bed porosity and particles size, distribution, shape and particles arrangement (these parameters assign the length of the path of the tracer through the bed characterized by the so called tortuosity), (ii) seepage velocity (linear [cm/s], pore rate of flow), viscosity and density of given liquid phase, (iii) dispersion and molecular diffusion coefficients corresponding to given tracer, (iv) interaction of the tracer with the solid phase, if exists, (because in a such case the so called effective or apparent dispersion coefficient, *D*_a_, is a function of the retardation coefficient, *D*_a_ = *D*_d_/*R*). Of course, the last point is out of question, if *R* converges to one. In sum, the following rule should be valid for the system studied: the smaller is the grain size, the greater is the *Pe*, or, by increasing the *Pe*, the plug (piston) flow is approached, in other words, the dispersion, i.e. the value of dispersion coefficient, is minimized.

**Fig. 5:**
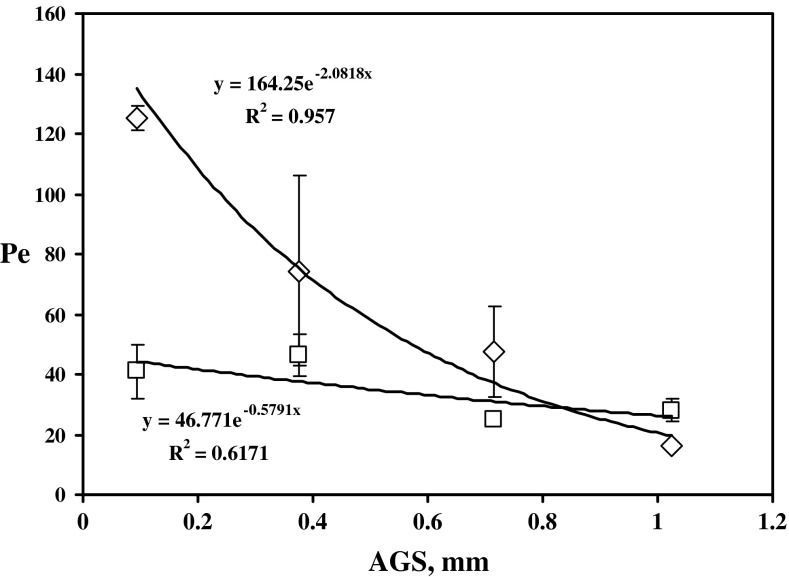
The dependences of Pe, calculated for sorption edges of HTO, on the average grain size (AGS) of crushed pure granite (*open diamond* ± SD) and infill material (*open square* ± SD) (*R* ^2^ is regression coefficient)

**Fig. 6:**
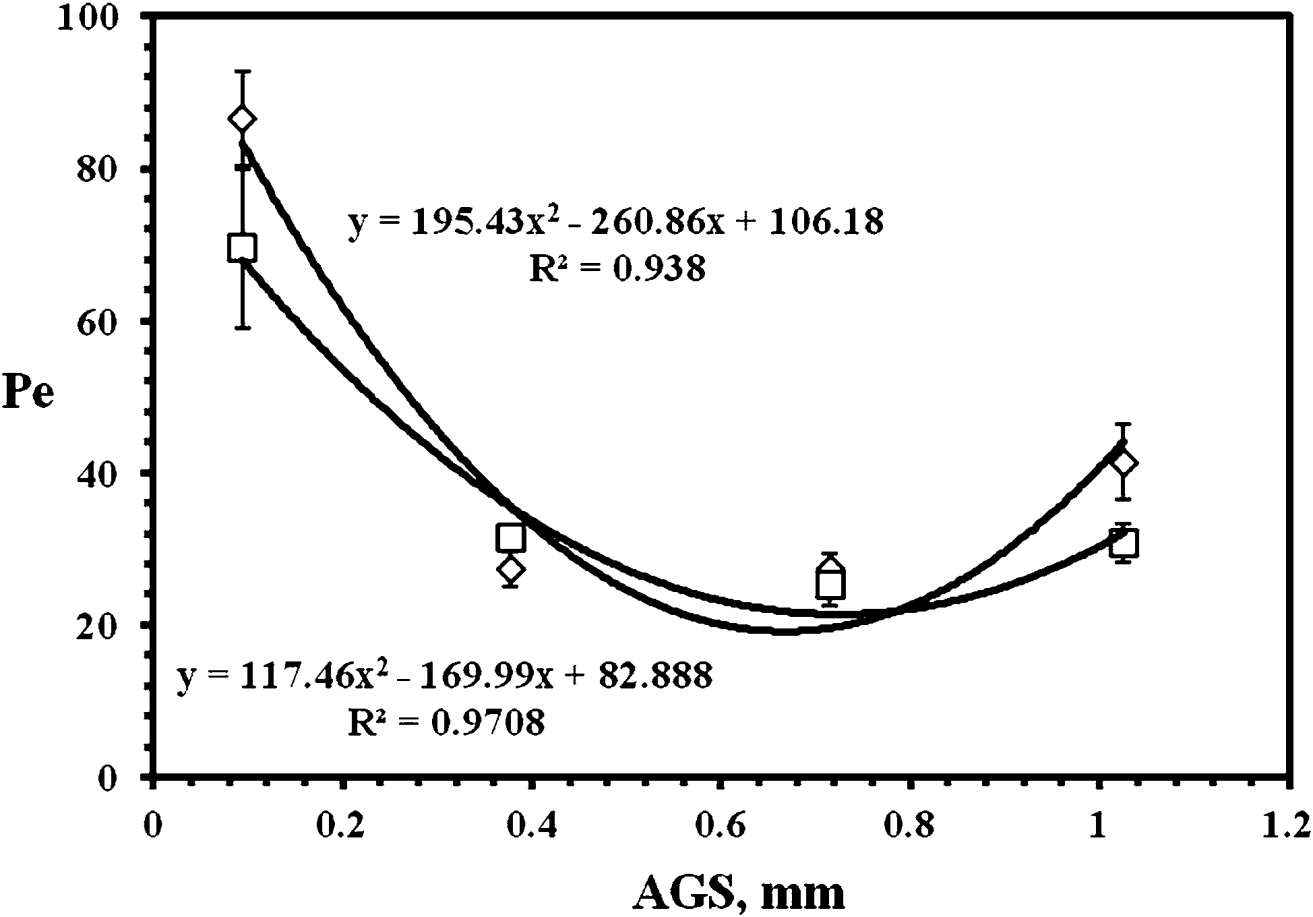
The dependences of Pe, calculated for sorption edges of ^36^Cl^−^ (as Na^36^Cl), on the average grain size (AGS) of crushed pure granite (*open diamond* ± SD) and infill material (*open square* ± SD) (*R* ^2^ is regression coefficient)

The graphical evaluation of *Pe*_S_ (corresponding to the sorption edge of BTC in column experiments, e.g., see Figs. 1, 3) is depicted in Fig. 5 (for HTO) and 6 (for ^36^Cl^−^). We see that the dependences in both figures (except of *Pe*_S_ values for the coarsest fraction in Fig. 6) in principal agree with the theory mentioned above. In addition, the regressions by means of exponential (Fig. 5) and polynomial (Fig. 6) function, having the acceptable values of regression coefficients, reflect the physical meaning of obtained dependences. Of course, the differences between behavior of HTO and ^36^Cl^−^ exist. Again, these differences are approximately comparable with the results obtained in [10] according to which: experiments with natural fracture column showed higher retardation and dispersion of tritiated water in comparison with chlorine probably due to ion-exclusion of the anions in fracture infill. From this point of view, if we compare the infill material values of *Pe*_S_ ± SD, summarized in Table 4; Fig. 5 (tracer HTO), and in Table 6; Fig. 6 (tracer ^36^Cl^−^ as Na^36^Cl), we see that the similar results were obtained, namely, there are lower Pe_S_ values (higher dispersion) of HTO in infill material columns, or worse approach to the plug flow, in comparison with values of ^36^Cl^−^ (except of column No. PDM1-2b, but if the values of Pe_S_ ± SD are taken into account, it approximately holds for this column, too).

The difference between both materials studied documents the fact that in a case of pure granite columns (especially see no. PDM1-1a–c in Table 4 or in Fig. 5) the higher values of *Pe*_S_ of tracer HTO were found, evidently as a result of the infill material absence. Regarding the column No. PDM1-1d, it seems that the most probable reason of the relatively higher dispersion of HTO, or small value of Pe_S_, consists either in the non-homogeneity of pure granite and in sampling of coarse fraction, or in the different tortuosity of the tracer path.

## Conclusion**s**

The experimental and theoretical values of transport parameters (*R*_*S*_ *, R*_*D*_ *, K*_dS_ *, K*_dD_, Pe_S_ and *D*_d_) were determined for HTO and ^36^Cl^−^ (as Na^36^Cl) transport in crushed pure granite and in its fracture infill material of different grain size. A series of dynamic experiments demonstrated similar behavior of studied radionuclides. With a few exceptions, the values of theoretical retardation coefficients were practically equaled 1 and the distribution coefficient values converged to zero in case of all fractions of crushed granite as well as of fracture infill material. This means that no noticeable interaction of these radionuclides was found with studied rock materials, only a very weak ion exclusion of ^36^Cl^−^ was observed in the case of infill material. Also, the influence of grain size on retardation coefficients was not found. Practically, tritium and chloride behaved as non-interacting, conservative tracers. Different pattern was observed in case of Pe and dispersion coefficient. Generally, Peclet numbers for tritium and chloride decreased (and logically dispersion coefficients increased) with increasing grain size of granite and infill material. But, the dependences did not agree with the theoretical suppositions in all cases. The differences could be caused, e.g., by the size and shape distribution of particles in bed, by the non-homogeneity of samples and the non-uniform bed porosity resulting in different tortuosity of the path of given tracer in the bed. Generally, the flow pattern can be different even if the column media originates from the one borehole.
